# New Access Routes to Undertreated Populations; How Do Problem Substance Users Recruited from an Unemployment Office Differ from Detoxification Treatment Inpatients?

**DOI:** 10.3390/ijerph182413014

**Published:** 2021-12-09

**Authors:** Norbert Scherbaum, Thomas Mikoteit, Lilia Witkowski, Udo Bonnet, Michael Specka, Fabrizio Schifano, Bodo Lieb

**Affiliations:** 1 LVR-Hospital Essen, Department of Addiction Medicine and Addictive Behavior, Medical Faculty, University of Duisburg-Essen, 45147 Essen, Germany; lilia.witkowski@web.de (L.W.); Michael.Specka@lvr.de (M.S.); 2 Job Center Essen, 45136 Essen, Germany; thomas.mikoteit@jobcenter.essen.de; 3 Evangelisches Krankenhaus Castrop-Rauxel, Klinik für Psychiatrie, Psychotherapie und Psychosomatische Medizin, 44577 Castrop-Rauxel, Germany; udo.bonnet@uni-due.de; 4 Psychopharmacology, Substance Misuse and Novel Psychoactive Substances Research Unit, School of Life and Medical Sciences, University of Hertfordshire, Hatfield AL10 9EU, UK; f.schifano@herts.ac.uk; 5 Katholisches Krankenhaus Hagen, Klinik für Psychiatrie and Psychotherapie, 58119 Hagen, Germany; b.lieb@kkh-hagen.de

**Keywords:** unemployment, treatment gap, addictive disorders, alcohol dependence, cannabis dependence

## Abstract

Background: Only a minority of subjects with substance use disorders (SUDs) are in addiction-specific treatment (treatment gap). Co-operation between an unemployment office and a psychiatric hospital was established for the assessment and counseling of long-term unemployed clients with SUD. We aim at validating whether such a treatment gap exists in that group, and whether clients from an unemployment office differed from a matched group of inpatient detoxification patients with regard to socio-economic characteristics, substance use and treatment history, and the prevalence of mental disorders Methods: Unemployment office clients (*n* = 166) with an SUD were assessed using a standardized sociodemographic and clinical interview. They were compared with 83 inpatients from a local detoxification ward, matched for age, sex, and primary addictive disorder (matching ratio 2:1). Results: Most (75.9%) subjects were males, with an average age of 36.7 years. The SUDs mostly related to alcohol (63.9%) and cannabis (27.7%). Although most unemployment office clients had a long SUD history, only half of them had ever been in addiction-specific treatment during their lifetime, and only one in four during the last year. There were no statistically significant differences between the groups regarding age at onset of problematic substance use, the proportion of migrants, and prevalence of comorbid mental disorders. The unemployment office sample showed lower levels of education (*p* < 0.001), job experience (*p* = 0.009), and current employment rates (*p* < 0.001). Conversely, inpatients showed lower rates of imprisonment (*p* < 0.001), more inpatient detoxification episodes (*p* < 0.03); and longer abstinence periods (*p* < 0.005). Conclusions: There was a lifetime and recent treatment gap in the group of long-term unemployed subjects with alcohol and cannabis dependence. The markedly lower educational attainment, chronic employment problems and higher degree of legal conflicts in the client group, as compared with patients in detoxification treatment, might require specific access and treatment options. The co-operation between the psychiatric unit and the unemployment office facilitated access to that group.

## 1. Introduction

Substance use disorders (SUD) are one of the major challenges for health care systems worldwide, as they show high prevalence levels, often take a chronic course, and are associated with both comorbid somatic/mental disorders and a high burden of social problems [1]. In Germany, the estimated prevalence of alcohol dependence in the adult population is 3.1% (*n* = 1.6 million); of cannabis dependence, 0.5% (*n* = 309,000) [2]; and of heroin dependence 0.3% (*n* = 170,000) [3]. However, SUD subjects, and especially those with alcohol-related disorders, show low rates of utilization of addiction-specific treatment services (treatment gap) [4]. It has been suggested that only about 15% of subjects with substance-related disorders (e.g., alcohol, nicotine, medication abuse/dependence) are in a substance-specific treatment at any given moment in Germany [5]. This treatment gap level cannot be explained by problems regarding the financing of treatment, as SUD treatment is covered in Germany by both statutory health insurances and pension funds. However, there might be other obstacles to starting an SUD treatment, including the stigmatization of both the SUD itself and of the related treatment institutions [6,7]. Hence, one could argue that additional ways to approach untreated subjects with SUD need to be considered.

According to international studies, unemployed persons have a higher prevalence of SUD than those in employment [8,9]. The SUNRISE-project (integrated support of unemployed at risk of substance abuse disorders) is a co-operation project between the Essen University Hospital addiction services and the local unemployment office; this is an institution responsible for the financial support, employment-related training, and job placement of local citizens, especially those with a long-term (>1 year) unemployment history. The co-operation aims at an improved provision of addiction-specific treatment to clients with SUDs.

By using treatment history data, the present study aims at identifying whether the existence of a treatment gap can be validated in this group. In addition, by comparing them with patients currently in SUD-specific treatment, we want to identify possible distinct characteristics the currently untreated client group.

## 2. Methods

### 2.1. Settings and Recruitment

In Germany, people with a long-term (e.g., >1 year) unemployment history receive financial support by the unemployment office. They remain in regular contact with the office, where they usually see a permanent case manager. The case manager checks the client’s financial needs; provides advice regarding further job education; and informs on available jobs. In Essen, a large city within a wide urban region in the Western part of Germany, a system of co-operation was agreed upon between the local unemployment office and the psychiatric department of the local university hospital; focus was on those long-term unemployed clients whose case manager was of the impression that the presence of underlying substance-related problems was a relevant factor behind the persistence of unemployment (for a detailed description of the SUNRISE project, see [10]). In the case of suspected substance-related problems in clients younger than 50, the case manager commissioned here the psychiatric hospital to carry out a professional assessment of the index client, focusing on the possible existence of a substance use and/or a comorbid mental disorder; possible treatment options; and the ability to work. The assessment was carried out in three sessions, with the first one being carried out at the unemployment office and the following sessions in the hospital to facilitate the possible up-take of a related addiction treatment; participation was voluntary.

Based on a previous analysis of the SUNRISE-project, it was here expected that most frequent SUD among long-term unemployed clients were alcohol- and cannabis-related disorders [10]. Accordingly, subjects for the comparison group were recruited from an in-patient detoxification ward specializing in alcohol and cannabis dependence. The inpatient treatment duration was here up to three weeks and consisted of a full diagnostic process; a medical treatment to both reduce withdrawal symptoms and coping with comorbid mental disorders; and the provision of social support to organize further treatment. This involved transfer either to a long-term inpatient abstinence-oriented treatment facility or to an outpatient treatment. The detoxification ward is part of a university department of addiction medicine which also includes a detoxification ward for opiate addicts, two opiate-substitution treatment clinics, and an outpatient service for patients with alcohol- and cannabis-related disorders. All patients younger than 50 years of age were invited to participate. Exclusion criteria were: insufficient command of the German language; lack of provision of informed consent; and presence of an acute psychiatric illness, e.g., schizophrenic psychosis.

### 2.2. Study Design

Clients of the unemployment office diagnosed with an SUD were compared with patients from an inpatient detoxification ward mainly for persons with alcohol dependence and cannabis dependence. Patients had been self-referred to treatment or referred by a general practitioner. Main variables for comparison between the two groups included: a history of SUD; a history of SUD treatment and/or other mental disorders; social status; professional history. For each detoxification patient included in the study, two matching subjects (e.g., in terms of sex, age (+/− 3 years), and main substance of abuse, excluding tobacco) from the SUNRISE project were here identified and selected for participation.

### 2.3. Assessments

All recruited subjects, from both the hospital and the unemployment office, were interviewed using the German version of the European Addiction Severity Index (EuropASI) [11], and the German version of the M.I.N.I.-SKID-I for the diagnosis of mental disorders (apart from personality disorders) according to DSM-IV [12].

### 2.4. Statistical Analyses

For categorical variables (e.g., sex, diagnosis) either the Chi² or Fisher’s exact test, as appropriate, was here carried out, whilst for ordinal or interval scaled variables (e.g., age, length of lifetime drug use) the Mann–Whitney U test was used. Due to the large number of statistical tests, in order to decrease a possible accumulation of type 1 errors a significance level of *p* < 0.01 (e.g., instead of *p* < 0.05) was here considered.

### 2.5. Ethics

Participation in the study was voluntary, and all subjects gave their written consent. Ethical approval was granted by the ethics committee of the University Hospital Essen (file number 15-6263-BO).

## 3. Results

During the 13-month data collection period, 269 different patients younger than 50 were admitted to the detoxification ward. Eight patients could not participate in the study due to severe difficulties with the command of the German language; seven could not be interviewed due to severe comorbid, including severe psychotic, disorders; 11 had to be moved to other departments due to comorbid somatic diseases before the interview could take place; 77 could not be interviewed due to their premature discontinuation of the inpatient treatment; and 63 declined to participate. Hence, 103/269 patients were here fully interviewed.

In the process of carrying out proper matching procedures, some 327 unemployment center clients younger than 50 were assessed within the framework of the SUNRISE project and received a diagnosis of a substance use disorder, excluding nicotine dependence. However, since in the initial clinical sample there was both a higher proportion of cannabis dependence subjects (e.g., 31% versus 14.1%), and a higher proportion of females (30% versus 20.5%) than in the initial SUNRISE sample, 20 inpatients could not be matched. Hence, a final number of 83 inpatients were properly matched to 166 SUNRISE unemployment project clients.

Both groups consisted mainly of middle-aged males (75.9%; clinical sample 36.6. years; SUNRISE sample: 36.8). Main SUD diagnoses for both groups were alcohol (63.9%) and cannabis dependence (27.7%) (Table 1). Regarding sociodemographic characteristics, the unemployed sample presented with a significantly lower level of education (*p* < 0.001), including a higher rate of incomplete school education (*p* = 0.01). In addition, unemployment office clients significantly more often reported having been previously inflicted with a prison sentence (*p* < 0.001). The remaining sociodemographic variables (e.g., migrant background, current partnership) showed no statistically significant group differences. In both groups, only a minority were currently living in a stable partnership and/or with children (see Table 1).

Regarding history of substance use, excluding nicotine, there were no significant differences regarding the initiation age of: regular substance use; problematic substance use; and regular use of the main problematic substance. Both groups had started regular substance use at an average age of around 20 years. In contrast, the clinical sample had been more successful regarding levels of intermittent achievement of abstinence, e.g., there was a higher percentage of subjects who had ever been abstinent for at least three months (56.6% vs. 38.6%; *p* = 0.007; see Table 2).

The clinical sample had more lifetime experience of addiction-related treatment, including both inpatient (*p* = 0.03) and outpatient (*p* < 0.001) treatment (see Table 3). In both groups, only a quarter had ever undergone a long-term rehabilitation treatment; only a small minority in both groups had ever participated in a self-help group. About one quarter of SUNRISE clients, but nearly half of the detoxification patients, had used specialized drug treatment services during the previous 12 months (*p* = 0.002).

In both groups, a lengthy and systematic procedure was carried out to diagnose a possible mental disorder in the study subjects. About half of the participants in both samples were affected by an affective disorder (depression or dysthymia, Table 4). While this differed not significantly between groups, the proportion of subjects with an anxiety disorder or PTSD was significantly larger in the SUNRISE group (26.2% versus 11.0%). In both groups, only a small number of subjects affected by a psychotic disorder were identified (ward: 1.2%; SUNRISE: 2.1%).

## 4. Discussion

Nearly half of the long-term unemployed clients of the SUNRISE project, who were identified with having a substance use disorder (SUD), had never been in addiction-specific treatment. This was despite the fact that they had started problematic substance use years ago. Moreover, three out of four clients had not been in treatment during the previous year. For the group of SUNRISE clients, therefore the existence of a lifetime and recent treatment gap can be validated.

Although there was no marked difference in the in-patient treatment group, matched by gender and age, with regard to age at initiation of problematic substance use, the patients had been in addiction-specific inpatient or outpatient treatment at much higher rates, and had been more successful regarding levels of intermittent achievement of abstinence, e.g., there was a higher percentage of subjects who had ever been abstinent for at least three months. They had also been in psychiatric treatment more often.

As a group, the SUNRISE clients had about the same low rate of stable partnerships and of living with children as the patient group, but they had much more chronic unemployment (by definition, regarding the target group of the project), less education, and many more legal conflicts. How can this group be motivated for treatment and be accessed by treatment providers?

The treatment gap [13] is considered a serious problem in mental health care, especially in the treatment of substance use disorders. Although psychotherapeutic and pharmacological treatments with documented effectiveness are available, and especially so for alcohol dependence [14,15]), only a minority of affected subjects is currently in addiction-specific treatment [5]. A number of factors behind the treatment gap have been identified, including the individual’s socio-economic status [16]; his/her emotional issues, such as shame and problem denial; or a lack of knowledge about possible treatment options. Conversely, the existence of service providers’ waiting lists might play a role as well.

A number of strategies have been developed to deal with the treatment gap for substance use disorders, e.g., implementation of awareness campaigns, including information on treatment options and prognosis, and improvement of the cooperation between the general health care system and addiction medicine. In this respect, the training of general practitioners in motivational interviewing whilst improving their knowledge about the local addiction services to increase their levels of patient referrals, is an option which has been considered [17]. Another strategy being suggested is to remove the barriers to addiction services whilst establishing clinical contact with SUD subjects outside the traditional services, e.g., at the workplace [18]; in prison [19]; in medical outpatient clinics [20]; and as was the case here, at an unemployment office. Whether these multiple contact strategies will better facilitate the clients’ uptake of long-term addiction-related treatments is a matter for further studies.

### Limitations

The current investigation relied on self-reports, which may be subject to both conscious (e.g., providing the most acceptable answers only) and memory-related distortion issues. Conversely, there might be no reason to believe that false reports were more represented in one group over the other. One would argue about the appropriateness of comparing SUD clients recruited at an unemployment unit with SUD inpatients, who may not necessarily be representing the whole population of SUD subjects. However, detoxification wards in Germany have a strategic importance for the implementation of any abstinence-oriented treatment. Indeed, outpatient detoxification is an uncommon practice, as documented here as well; this approach was in fact mentioned by only 5% of subjects from both groups during their lifetime, compared with a 47% rate for inpatient detoxification episodes even in the SUNRISE sample. In addition, a successfully completed detoxification treatment is often required as a precondition for a long-term, abstinence-oriented, either outpatient or rehab, treatment being offered to alcohol- or cannabis-related disorder clients. Hence, detoxification ward inpatients may well be representative, in Germany at least, of those who enter an addiction-specific treatment package. Finally, 61.7% of the detoxification ward inpatients were not included here, because either premature treatment discontinuation or refusal to participate, possibly associated to the lengthy (e.g., 2–3 sessions, taking up 4 h) assessment procedures. Conversely, without a systematic and thorough diagnostic procedure, a credible comparison between these two groups could not have taken place.

## 5. Conclusions

For long-term unemployed clients an SUD treatment gap was observed. Further interventions should be developed and evaluated to increase their motivation to start addiction-specific treatment.

## Figures and Tables

**Table 1:** Comparison of sociodemographic characteristics comparing clinical sample (ward; *n* = 83) and unemployment office sample (SUNRISE; *n* = 166).

		Ward	SUNRISE	*p*
Gender	male	75.9%	75.9%	
	female	24.1%	24.1%	
Age	mean (SD)	36.6 (7.8)	36.8 (7.7)	
	median (min–max)	36 (25–48)	36 (24–49)	
Main Drug	alcohol	63.9%	63.9%	
	cannabis	27.7%	27.7%	
	benzodiazepines	2.4%	2.4%	
	stimulants	2.4%	2.4%	
	opiates	3.6%	3.6%	
Migration background		13.3%	18.8%	0.27
School not completed		10.8%	24.7%	0.01
Median educational level (9 levels) ^1^		5	3	<0.001
Vocational training/study completed		55.4%	45.2%	0.13
Ever employed		88.0%	73.5%	0.009
Mostly unemployed during last 3 years		39.0%	83.6%	<0.001
No current employment or training		54.2%	87.2% ^2^	<0.001
Total length of employment (years)	mean (SD)	7.6 (7.2)	3.9 (5.3)	<0.001
	median (min–max)	5 (0–30)	2 (0–23)	
Longest duration of employment (years)	mean (SD)	4.8 (5.2)	3.3 (4.4)	0.001
	median (min–max)	3 (0–26)	32 (0–23)	
Duration of current unemployment	mean (SD)	3.1 (5.2)	6.0 (6.0)	<0.001
	median (min–max)	0.7 (0–25)	7 (0.1–30)	
Stable partnership	yes	26.5%	27.4%	0.88
Living with children	yes	14.8%	10.5%	0.33
Main company during leisure time	alone	39.8%	40.1%	*p* = 0.10
	relatives/friends with drug/alcohol problems	26.5%	29.0%	
	people without drug or alcohol problems	33.7%	39.9%	
Ever delinquent	yes	63.8%	65.1%	0.83
Ever sentenced to prison	prison sentence	7.2%	33.3%	<0.001
	probation only	19.3%	4.9%	
	none	73.5%	61.7%	
Currently on probation	yes	8.4%	8.3%	0.97
Months in prison	mean (SD)	5.6 (19.3)	6.2 (18.3)	0.10
	median (min–max)	0 (0–156)	0 (0–144)	

^1^ two difficult to evaluate foreign degrees in each group not included. ^2^ 12.8% had either a current part-time job, with a monthly income of less than 400 Euros, or carried out low payment, public interest, work (“1-Euro-job”).

**Table 2:** Prevalence of substance use, excluding nicotine; comparison between clinical sample (inpatient ward) and unemployment office sample (SUNRISE).

		Ward	SUNRISE	*p*
Number of regularly consumed substances (lifetime)	mean (SD)	2.5 (1.3)	2.2 (1.4)	0.03
	median (min–max)	2 (1–5)	2 (0–7)	
Age at start of any regular substances use	mean (SD)	19.2 (5.8)	20.4 (8.3)	0.95
	median (min–max)	18 (8–42)	17 (8–47)	
Age at start of problematic substance use	mean (SD)	19.4 (6.7)	20.4 (9.5)	0.85
	median (min–max)	18 (8–42)	18 (8–47)	
Number of substances used recently ^1^	mean (SD)	2.0 (1.2)	1.4 (1.0)	<0.001
	median (min–max)	2 (1–5)	1 (0–5)	
Number of substances used 4 or more times recently ^1^	mean (SD)	1.6 (0.9)	1.2 (0.8)	<0.001
	median (min–max)	1 (0–5)	1 (0–5)	
Age at start of regular use of main problem substance	mean (SD)	21.5 (7.2)	22.1 (8.3)	0.95
	median (min–max)	20 (11–43)	19 (8–45)	
Age at start of problematic use of main problem substance	mean (SD)	23.3 (7.7)	22.4 (9.1)	0.16
	median (min–max)	23 (12–43)	21.5 (8–45)	
Ever abstinent from main substance for at least 3 months	Yes	56.6%	38.6%	0.007
Longest period of abstinence in months (none = 0)	mean (SD)	11.3 (22.5)	6.3 (11.1)	0.005
	median (min–max)	4 (0–120)	0(0–60)	

^1^ during the last 30 days, or the last 30 days before admission to current treatment, respectively.

**Table 3:** Treatment history; comparison between clinical sample (inpatient ward) and unemployment office sample (SUNRISE).

		Ward	SUNRISE	*p*
Out-patient detoxification; lifetime	yes	4.8%	6.6%	0.57
Previous in-patient detoxification treatment; lifetime	yes	61.4%	47.0%	0.03
Any lifetime detoxification treatment	yes	63.9%	50.6%	0.048
Number of previous inpatient detoxification treatments	mean (SD)	3.7 (7.4)	3.3 (10.4)	0.04
	median (min–max)	1 (0–45)	1 (0–110)	
Age at first detoxification treatment	mean (SD)	31.2 (7.4)	31.9 (8.1)	0.59
	median (min–max)	31 (18–48)	31 (10–48)	
Long-term residential treatment, lifetime	yes	29.6%	25.5%	0.49
Age at first long-term residential treatment (inpatients *n* = 26; SUNRISE *n* = 40)	mean (SD)	31.6 (7.2)	33.7 (6.2)	0.22
	median (min–max)	31.5 (21–46)	33 (24–48)	
Number of long-term residential treatments	mean (SD)	0.68 (1.3)	0.44 (1.0)	0.28
	median (min–max)	0 (0–6)	0 (0–7)	
Out-patient treatment; lifetime	yes	24.1%	9.0%	0.001
Self-help group attendance; lifetime	yes	13.3%	8.4%	0.23
Addiction treatment provided by a psychiatrist; lifetime	yes	1.2%	6.0%	0.11
Assisted living; lifetime	yes	3.6%	10.2%	0.07
Any previous specialized addiction treatment ^1^; lifetime	yes	65.1%	53.6%	0.085
Specialized addiction treatment in the last year	yes	46.3%	26.4%	0.002

^1^ Detoxification, opiate maintenance treatment, out-patient addiction clinic, or residential treatment.

**Table 4:** Current comorbid mental disorders and history of psychiatric treatment; comparison between clinical sample (inpatient ward) and unemployment office sample (SUNRISE).

	Ward	SUNRISE	*p*
**Main DSM-IV Diagnoses ^1^**			
Psychotic disorder	1.2%	2.1%	0.98
Depressive disorder	50.0%	37.7%	0.07
Dysthymic disorder	4.8%	12.2%	0.17
Anxiety disorder; PTSD	11.0%	26.2%	0.007
**Psychiatric treatment (lifetime)**			
Psychiatric out-patient treatment lifetime	42.2%	26.4%	0.012
Psychiatric in-patient treatment lifetime	23.5%	17.7%	0.01
Any psychiatric treatment lifetime	56.6%	35.6%	0.002
Psychopharmacological medication lifetime	57.3%	27.8%	<0.001
Age at first psychiatric treatment (mean, SD)	28.8 (11.4)	25.8 (10.6)	0.29

^1^*n* = 82 inpatients, *n* = 144 (SUNRISE clients); reduced sample size due to participants missing the second appointment.

## Data Availability

Data available on request.
